# Assessing European Union member states’ progress toward antimicrobial sales reduction targets under the European Green Deal: A comparative policy and performance analysis

**DOI:** 10.14202/vetworld.2025.2746-2760

**Published:** 2025-09-18

**Authors:** Aina Muska, Irina Pilvere, Ilze Upite, Kristaps Muska, Aleksejs Nipers

**Affiliations:** Faculty of Economics and Social Development, Latvia University of Life Sciences and Technologies, Jelgava, Latvia

**Keywords:** antimicrobial resistance, antimicrobial sales, EU Member States, European Green Deal, Farm to Fork strategy, policy performance

## Abstract

**Background and Aim:**

Antimicrobial resistance (AMR) is a growing One Health threat driven by the excessive use of antimicrobials (AMs) in human and veterinary medicine. Recognizing this, the European Green Deal’s Farm to Fork (F2F) strategy set a target to reduce veterinary AM sales by 50% by 2030 compared to 2018 levels. Understanding the performance of European Union (EU) Member States (MS) is critical to evaluate progress and identify gaps. This study assesses the performance of EU-27 MS in reducing veterinary AM sales during the progress period (2018–2022) and estimates their likelihood of achieving the 2030 reduction target.

**Materials and Methods:**

Data from the European Medicines Agency (European Surveillance of Veterinary Antimicrobial Consumption) and Common Agricultural Policy (CAP) Strategic Plans were analyzed. Indicators included AM sales (mg/population correction units [PCUs]), trends from 2018–2022, and national targets for CAP Strategic Plans result indicator R.43 (“Limiting antimicrobial use”). MS were grouped by performance level, strong, average, limited, or insufficient, using the zero unitarization method, and results were visualized with color-coded classifications.

**Results:**

Between 2018 and 2022, AM sales for food-producing animals declined by 31% across the EU, with notable reductions in Portugal (−57%), Malta (−48%), France (−44%), and Latvia (−43%). However, sales increased in Poland (+7%) and Lithuania (+36%), and the largest absolute sales remained concentrated in Spain, Poland, Italy, and Germany (over 70% of total EU sales). PCU analyses revealed uneven intensity of AM use, with Spain and Poland showing high usage rates, while Germany and France demonstrated more efficient use. During the progress period, 20 MS were rated as strong contributors, while three MS showed limited contributions. For the target period (2023–2030), only six MS (e.g., Italy, Romania, Latvia, and Malta) are projected to maintain strong contributions, whereas seven MS, including Poland, Lithuania, and Denmark, are unlikely to achieve the target.

**Conclusion:**

The EU-27 achieved substantial reductions in AM sales during 2018–2022, yet performance remains uneven across MS. While some countries have already met or are close to the 2030 target, others require accelerated policy interventions, veterinary stewardship, and stronger CAP alignment. Achieving the EU-wide 50% reduction by 2030 will depend heavily on high-consumption MS adopting best practices from leading countries.

## INTRODUCTION

Antimicrobials (AMs) are indispensable for treating and controlling infectious diseases in both humans and animals. However, their extensive and often inappropriate use has raised significant concerns about AM resistance (AMR), a major global public health threat [1]. Resistant pathogens can be transmitted from animals to humans, complicating treatment outcomes and increasing morbidity and mortality rates [2]. The rise of AMR undermines the effectiveness of bacterial infection treatments, as many microorganisms have developed resistance to multiple drugs [3]. Recognizing this, the World Health Organization officially declared AMR a global health threat in 2014 [4] and introduced the Global Action Plan in 2015 to curb its spread by promoting disease prevention and optimizing antibiotic use in both human and veterinary medicine [5].

The European Union (EU) has also taken steps by regulating veterinary AM products, prohibiting their use for growth promotion in imported animals, and restricting antibiotics that are critical for human medicine [6]. To address AMR comprehensively, the One Health approach, integrating human, animal, and environmental health, is widely applied, aiming to safeguard the long-term efficacy of AMs by minimizing unnecessary use [7]. Within this framework, reducing veterinary antibiotic consumption is emphasized [8], as more than half of AMs are used inappropriately across human and veterinary sectors [9]. Veterinarians, therefore, play a pivotal role in optimizing AM use in farm animals [10].

In livestock production, AMs are frequently administered for disease treatment, yet entire herds are sometimes treated preventively, blurring the distinction between therapeutic and prophylactic use [11]. While disease management in livestock is essential to reduce morbidity, mortality [8], and economic losses [12], reliance on antibiotics must be limited. Alternative disease control strategies, along with the reduction or prohibition of subtherapeutic AM use as growth promoters, are urgently needed [13, 14]. Without decisive action, unchecked AM consumption is projected to cause cumulative global economic losses of up to USD 100 trillion by 2050 [9].

To counter this, innovative strategies for AM stewardship (AMS) are required, alongside broad stakeholder engagement to ensure sustainable AM use [15]. Promoting responsible AM practices in agriculture has become an EU priority [16]. Within the European Green Deal (2019), the Farm to Fork (F2F) strategy seeks to transform the food supply chain into a more sustainable system. A key target is to halve AM sales in livestock and aquaculture by 2030 compared with 2018 levels, reducing EU sales from 118.4 mg/kg population correction units (PCUs) in 2018 to 59.2 mg/kg PCU by 2030 [17]. Correspondingly, the European Commission has set country-specific targets for all EU Member States (MS), requiring each to reduce AM sales per kg PCU by 50% relative to their 2018 baseline [18].

Despite extensive recognition of AMR as a global health crisis, there remains a limited body of comparative evidence on how individual EU MS are progressing toward the specific AM reduction targets set under the F2F strategy of the European Green Deal. Most existing research emphasizes the biological mechanisms of AMR, the risks of inappropriate AM use, or broad regulatory frameworks at the EU level. However, relatively few studies provide a systematic performance assessment of MS against the quantifiable goal of a 50% reduction in veterinary AM sales by 2030. Moreover, available evaluations tend to focus either on aggregated EU-wide data or national-level interventions without sufficiently addressing differences in baseline consumption, the varying intensity of livestock production, and the alignment of national Common Agricultural Policy (CAP) Strategic Plans (SPs) with the EU target. There is also a gap in linking sales trends with PCU-based indicators that reflect the intensity of use relative to livestock biomass, as well as in forecasting future performance beyond the progress period (2018–2022). This lack of disaggregated, comparative, and forward-looking analysis creates uncertainty regarding which MS are likely to meet, exceed, or fall short of the 2030 targets, and what policy adjustments may be required.

To address this gap, the present study aims to evaluate the contributions of EU MS in achieving the European Green Deal’s F2F strategy objective of halving veterinary AM sales by 2030 compared with 2018 levels. Specifically, the study analyzes sales data reported by the European Medicines Agency (EMA) alongside national CAP SP indicator (R.43: “Limiting antimicrobial use”) to assess both past progress (2018–2022) and projected performance (2023–2030). MS are classified into groups based on their relative performance, taking into account sales intensity (milligrams per PCU [mg/PCU]), historical reduction rates, and future policy commitments. By comparing national-level trends with EU averages and identifying strong, average, limited, and insufficient contributors, the research provides evidence-based insights into the uneven progress among MS. The study ultimately seeks to highlight where policy interventions, veterinary stewardship, and cross-country exchange of best practices are most urgently needed to ensure the EU-wide achievement of the 2030 AM reduction target.

## MATERIALS AND METHODS

### Ethical approval

No ethical approval was required for this research as it exclusively utilized secondary, aggregated information from publicly available datasets.

### Study period and location

The study was conducted from October 1, 2024, to July 1, 2025, in Latvia.

### Research data

The research used data collected by the EMA from EU MS [19]. The study used the following indicators obtained from the EMA: (1) The sales of antibiotic agents as veterinary medicinal products marketed mainly for food-producing animals; (2) the PCUs of veterinary AMs relative to the animal population of European farms; and (3) the overall sales of veterinary AMs measured in mg/PCU [19]. The PCU includes only food-producing animals, including horses and farmed fish, and this indicator considers the total population of each farmed animal species multiplied by the estimated average liveweight when treated with antibiotics.

The indicator sales of veterinary AMs (mg/PCU) is based on the framework defined by the European Commission within the context of the European Green Deal and the F2F strategy. According to Annexure 1 of the European Commission’s Recommendations to the Member States as Regards their Strategic Plan for the Common Agricultural Policy (2020) [20], this indicator is officially used to quantify progress toward the specific target of reducing the sales of AMs intended for food-producing animals and aquaculture by 50% by 2030.

This indicator is particularly suitable for assessing the situation in the agricultural sector, as it allows for the comparison of data across countries and periods by considering livestock population size. This ensures a standardized and internationally comparable approach. Moreover, its use is essential for evaluating the effectiveness of measures aimed at reducing AMR, which remains one of the most pressing public health challenges both within Europe and globally.

The indicator sales of veterinary AMs (mg/PCU) corresponds to the result indicator R.43, which is part of the Monitoring and Evaluation Framework of the CAP SPs. Indicator R.43, titled “Limiting antimicrobial use,” is directly aligned with the EU’s F2F strategy goal of reducing AM sales for farmed animals and aquaculture by 50% by 2030 [21]. It serves as the primary quantitative indicator for monitoring progress toward this target. Indicator R.43 measures the share of livestock units (LUs) affected by supported actions to limit AM use (prevention/reduction). The average target value for the EU-27 is 23.2% [21]. This means that almost one-quarter of the total LUs in the respective MS will be offset by CAP support payments to ensure that this target is achieved. As a standardized metric, indicator R.43 enables the assessment of actual changes not only at the national level but also within the EU’s broader context of agricultural practices. It also facilitates comparability between MS and over time, accounting for variations in livestock population size.

### Data sources

The research was based on information and data from the EMA [19] and the European Commission [21, 22].

### Data coverage limitations

Several limitations must be considered in this study. The European Surveillance of Veterinary Antimicrobial Consumption (ESVAC) data cover only the sales of officially registered and marketed veterinary AM agents, excluding off-label use and unofficial imports, which may affect the overall consumption assessment [19]. This limitation may impact the accuracy of the actual consumption estimate.

#### Data availability and reporting consistency

Although the study relied on official reports and publicly available data, not all EU MS provided equally detailed, complete, or up-to-date information on the sales volumes of veterinary AMs. At the beginning of the research period, the quality and completeness of sales data for veterinary AMs varied between countries. In some MS, data submission was based on voluntary participation rather than legal obligation. Furthermore, not all countries provided disaggregated data by AM substance group or animal species [19].

Only after the entry into force of Regulation (EU) 2019/6 in 2022 [23] did reporting of veterinary AM usage data become mandatory and progressively harmonized across all EU MS. This development significantly affected the quality and comparability of data.

The veterinary AM sales data used in this research, sourced from ESVAC project, were not adjusted to account for differences in reporting practices across countries or periods. Therefore, the results should be interpreted with caution, considering possible inconsistencies in data quality and completeness [19].

#### Assumptions in the change rate projections

This research calculated the observed average annual rate of change in AM sales from 2018 to 2022 (the progress period) and compared it with the required yearly average rate of change from 2023 to 2030 (the target period) to achieve politically set goals. This approach assumes that the historical rate of change will be maintained in the future. However, various factors, including policy changes, regulatory effectiveness, industry adaptation capabilities, and the economic environment, may influence future trends. The results, therefore, depict potential trajectories rather than precise forecasts.

### Research period

The study covers the period from 2018 to 2030. This period is divided into two parts to evaluate the performance of EU MS: the progress period (2018–2022) and the target period (2023–2030). The research period began in 2018, when the European Commission initiated monitoring of progress toward the objectives outlined in the F2F strategy. The most recent publicly available data on veterinary AM sales in EU MS refer to 2022. The research period ends in 2030, corresponding to the target year set by the European Commission to achieve a 50% reduction in AM sales in the EU.

The first section of the results analyzes the first two indicators over the progress period (2018–2022) to describe the overall situation and the changes in the EU and its MS. The second and third sections analyze the third indicator used by the EMA to assess the contributions of EU MS during the progress period (2018–2022) and in the target period (2023–2030).

### Grouping the EU MS by performance

To assess the current performance of the EU-27 in achieving the target of a 50% reduction in AM sales for farmed animals and aquaculture by 2030, the MS were grouped based on two differentiation indicators: (1) AM sales in 2022 (mg/PCU) and (2) AM sales for farmed animals and aquaculture (%) over the progress period (2018–2022). The rate of change from the base year (increase/decrease, %) was calculated using Equation 1:


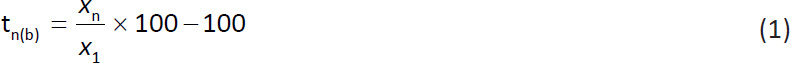


In Equation 1, t_n(b)_ is the rate of change from the base year (%), x_n_ is the indicator value at the end of the progress period, and x_1_ is the indicator value at the beginning of the progress period.

For the target period, EU MS were grouped using two differentiation indicators: (1) the average annual rate of change over the target period and (2) result indicator R.43 set by the CAP SP of the respective EU MS for 2023–2027, which represents current policies, future lines of action, and national governments’ political ambitions in achieving the target. Although the programming period ends in 2027, the implemented measures’ impacts will be visible until 2030.

Since the indicator-based trends observed in previous years do not indicate the current and future contexts but only the past context [24], the average annual rate of change was calculated for the statistical indicator both for the progress period (2018–2022) and for the target period (2023–2030) to identify the distance to the target. The average rate of change (%) indicates the average development intensity of the examined phenomenon, representing the frequency at which average levels of the time series change over time. It was calculated using Equation 2 as follows:


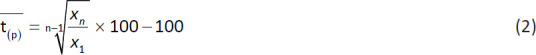


In Equation 2, is the average annual rate of change (increase/decrease in %), x_n_ is the time-series series indicator value at the end of the period, x_1_ is the time-series series indicator value at the beginning of the period, and n is the number of time-series series levels.

To determine whether the pace of change should be increased, decreased, or maintained in the future, the average annual rate of change over the target period (B) was compared with the average annual rate of change over the progress period (A). A potential increase in the pace in the future would necessitate changes in current policies and actions to be taken by the respective MS.

The following estimate was made to interpret the required rate of change: For (A− B) < −4%, the achievement is considered probable; for −4% ≤ (A − B) < −2%, it is feasible; for −2% ≤ (A − B) < 0%, it is uncertain; for 0% ≤ (A − B) < 2%, it is difficult; for 2% ≤ (A − B) < 4%, it is very difficult; and for (A − B) ≥ 4%, it is improbable [23]. The difference (A − B) indicates the required change in the annual growth rate (in percentage points). Detailed definitions of A and B are provided in the Results section (Tables 1–4) [19, 22].

**Table 1:** EU MS included in Group 1 based on the required average annual change rate and result indicator R.43 set by the MS’s CAP SP for the target period [19, 22].

MS	Targets for R.43, %	Average annual growth rate from 2018 to 2022 (A), %	Required average annual growth rate from 2023 to 2030 (B), %	Required change in the annual growth rate (A−B), percentage points	Rating of achievement of the target	Aggregated indicator
RO	90.41	−12.35	−2.05	−10.31	Probable	0.832
IT	68.80	−10.37	−3.14	−7.22	Probable	0.683
MT	60.00	−16.55	0.38	−16.93	Target achieved	0.746
LV	56.44	−12.75	−1.83	-10.93	Probable	0.660
CY	55.79	−10.24	−3.21	−7.02	Probable	0.612
HU	51.84	−11.41	−2.58	−8.83	Probable	0.612
Average						**0.691**
Color legends for the indicator “Targets for R.43”:						
The indicator is above the EU average			The achieved figure is equal to the EU average (± 15%)		The indicator is below the EU average	

Red indicates negative values (decrease). Black indicates positive values (increase). Green highlights countries that have achieved the target.

EU = European Union, MS = Member State, CAP = Common Agricultural Policy, SP = Strategic Plans, RO = Romania, IT = Italy, MT = Malta, LV = Latvia, CY = Cyprus, HU = Hungary

**Table 2:** EU MS included in Group 2 based on the required average annual change rate and result indicator R.43 set by the MS’s CAP SP for the target period [19, 22].

MS	Targets for R.43, %	Average annual growth rate from 2018 to 2022 (A), %	Required average annual growth rate from 2023 to 2030 (B), %	Required change in the annual growth rate (A−B), percentage points	Rating of achievement of the target	Aggregated indicator
FI	98.10	−4.88	−5.98	1.10	Difficult	0.744
EL	49.30	−1.25	−7.72	6.47	Improbable	0.427
HR	28.90	−5.61	−5.61	0.00	Uncertain	0.392
SK	27.51	−4.40	−6.21	1.82	Difficult	0.364
CZ	24.37	−4.97	−5.93	0.96	Difficult	0.357
Average						0.457
Color legends for the indicator “Targets for R.43”:						
The indicator is above the EU average		The achieved figure is equal to the EU average (± 15%)			The indicator is below the EU average	

Red indicates negative values (decrease). Black indicates positive values (increase).

EU = European Union, MS = Member State, CAP = Common Agricultural Policy, SP = Strategic Plans, FI = Finland, EL = Greece, HR = Croatia, SK = Slovakia, CZ = Czech Republic

**Table 3:** EU MS included in Group 3 based on the required average annual change rate and result indicator R.43 set by the MS’s CAP SP for the target period [19, 22].

MS	Targets for R.43, %	Average annual growth rate from 2018 to 2022 (A), %	Required average annual growth rate from 2023 to 2030 (B), %	Required change in the annual growth rate (A−B), percentage points	Rating of achievement of the target	Aggregated indicator
AT	17.27	−7.85	−4.47	−3.37	Feasible	0.368
BE (Wallonia)	16.89	−10.19	−3.23	−6.96	Probable	0.407
PT	13.51	−19.48	2.19	−21.67	Target achieved	0.554
SI	7.88	−12.18	−2.15	−10.03	Probable	0.394
FR	7.77	−11.77	−2.37	−9.40	Probable	0.386
LU	7.20	−7.03	−4.89	−2.14	Feasible	0.301
BE (Flanders)	5.20	−10.19	−3.23	−6.96	Probable	0.345
ES	3.80	−12.67	−1.87	−10.79	Probable	0.381
IE	3.23	−7.50	−4.65	−2.85	Feasible	0.289
NL	not planned	−10.40	−3.13	−7.27	Probable	0.338
Average						**0.376**
Color legends for the indicator “Targets for R.43”:						
The indicator is above the EU average		The achieved figure is equal to the EU average (± 15%)			The indicator is below the EU average	

Red indicates negative values (decrease). Black indicates positive values (increase); green highlights countries that have achieved the target.

EU = European Union, MS = Member State, CAP = Common Agricultural Policy, SP = Strategic Plans, AT = Austria, BE = Belgium, PT = Portugal, SI = Slovenia, FR = France, LU = Luxembourg, ES = Spain, IE = Ireland, NL = Netherlands

**Table 4:** EU MS included in Group 4 based on the required average annual change rate and result indicator R.43 set by the MS’s CAP SP for the target period [19, 22].

MS	Targets for R.43, %	Average annual growth rate from 2018 to 2022 (A), %	Required average annual growth rate from 2023 to 2030 (B), %	Required change in the annual growth rate (A−B), percentage points	Rating of achievement of the target	Aggregated indicator
EE	22.72	−3.54	−6.63	3.09	Very difficult	0.324
PL	22.30	3.88	−10.03	13.91	Improbable	0.201
BG	17.64	−3.62	−6.59	2.97	Very difficult	0.299
SE	17.35	−3.25	−6.77	3.52	Very difficult	0.291
LT	16.24	10.19	−12.64	22.83	Improbable	0.069
DE	7.07	−5.70	−5.57	−0.13	Uncertain	0.278
DK	not planned	−2.54	−7.11	4.57	Improbable	0.205
Average						**0.238**
Color legends for the indicator “Targets for R.43”:						
The indicator is above the EU average		The achieved figure is equal to the EU average (± 15%)			The indicator is below the EU average	

Red indicates negative values (decrease). Black indicates positive values (increase).

EU = European Union, MS = Member State, CAP = Common Agricultural Policy, SP = Strategic Plans, EE = Estonia, PL = Poland, BG = Bulgaria, SE = Sweden, LT = Lithuania, DE = Germany, DK = Denmark

The indicator values achieved by EU MS were compared with the EU-27 average, which allowed grouping into four categories: (1) above the EU average and (2) below the EU average.

The assignment of group ranks was based on the aggregated indicator [25]. The indicators were standardized using the zero unitarization method [26], which allows comparison across different datasets on a uniform scale, represented as follows (Equations 3 and 4):


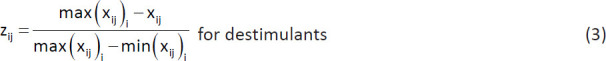



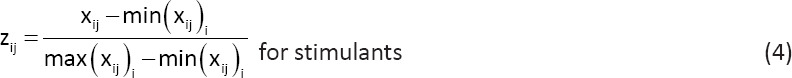


In Equations 3 and 4, z_ij_ is the normalized value of the j-th variable in the i-th MS, x_ij_ – is the initial value of the j-th variable in the i-th MS, min(x_ij_)_i_ – is the minimum value of x_ij_, max(x_ij_)_i_ – is the maximum value of x_ij_. In the mathematical model (Equations 3 and 4), the term “variable” refers to the raw and normalized values. Elsewhere in the text, these are referred to as “indicators”, reflecting their role in the evaluation framework. This method was chosen because it was the only one that met all seven postulates outlined for the procedure of standardizing indicator values [27].

Since normalized values ranged from 0 to 1, the average normalized value was calculated for each MS for both the progress and target periods, as described by Muska *et al*. [28]. Using these averages, the aggregated indicator value for each group was calculated, and a rank was assigned to each group. The closer the aggregated indicator is to 1, the higher the rank.

A color-coded visualization compared MS performance against the EU-27 average, showing whether an MS indicator value was higher, equal, or lower than the EU average (±15%). This threshold was selected as a robust criterion to account for statistical variability, measurement uncertainty, and cross-country differences, while remaining sensitive to substantive deviations [29]. Such thresholds are commonly applied in policy analysis [30] and comparative research [31].

The authors interpreted EU MS performance as follows:

- Strong contribution: Aggregated indicator >0.65.
- Contribution parity: Aggregated indicator 0.50–0.65.
- Limited contribution: Aggregated indicator 0.35–0.49.
- Insufficient contribution: Aggregated indicator <0.35.

Subsequently, MS contributions were compared between the progress and target periods to identify whether contributions (1) improved, (2) deteriorated, or (3) remained stable.

### Region examined

The research covered 27 EU MS: Austria (AT), Belgium (BE), Bulgaria (BG), Croatia (HR), Czech Republic (CZ), Cyprus (CY), Denmark (DK), Estonia (EE), Finland (FI), France (FR), Germany (DE), Greece (EL), Hungary (HU), Ireland (IE), Italy (IT), Latvia (LV), Lithuania (LT), Luxembourg (LU), Malta (MT), the Netherlands (NL), Poland (PL), Portugal (PT), Romania (RO), Slovakia (SK), Slovenia (SI), Spain (ES), and Sweden (SE). In the case of BE, separate CAP SP exist for Wallonia and Flanders; therefore, forecast indicators were assessed for each region independently.

## RESULTS

### Antibiotic agent sales and PCUs in the EU

**Figure 1:**
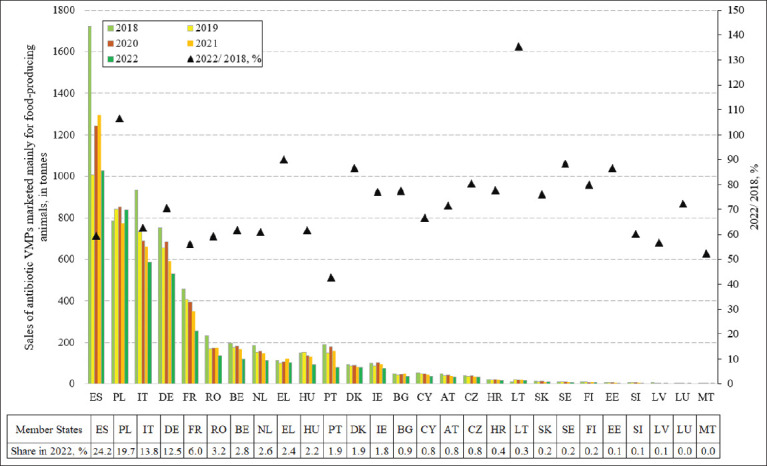
Sales of antibiotic veterinary medicinal products marketed mainly for food-producing animals in European Union Member States in 2018–2022, in tonnes, with 2022 share, % and change 2022 is 2018, % [19]. ES = Spain, PL = Poland, IT = Italy, DE = Germany, FR = France, RO = Romania, BE = Belgium, NL = Netherlands, EL = Greece, HU = Hungary, PT = Portugal, DK = Denmark, IE = Ireland, BG = Bulgaria, CY = Cyprus, AT = Austria, CZ = Czech Republic, HR = Croatia, LT = Lithuania, SK = Slovakia, SE = Sweden, FI = Finland, EE = Estonia, SI = Slovenia, LV = Latvia, LU = Luxembourg, MT = Malta.

Between 2018 and 2022, the EU-27 collectively reported a 31% decrease in sales of antibiotic agents for food-producing animals, declining from 6181 tonnes in 2018 to 4249 tonnes in 2022. During this period, the largest reductions were observed in PT (57%), MT (48%), FR (44%), LV (43%), RO (41%), and ES (40%), reflecting significant changes in government policies and practices. In contrast, LT and PL recorded increases of 36% and 7%, respectively. The increase in PL was particularly substantial, as it was the second-largest consumer of antibiotic agents in the EU, with sales rising by nearly 52 tonnes in 2022 compared with 2018 (Figure 1) [19].

In 2022 (Figure 1) [19], the largest sales were reported in ES (1027 tonnes), PL (838 tonnes), IT (585 tonnes), and DE (531 tonnes), which together accounted for over 70% of total EU sales. This suggests that these four MS will play a pivotal role in achieving the European Green Deal’s F2F strategy target of reducing AM sales by 50% by 2030. Notably, ES had already achieved a substantial reduction (−40%), as had IT (−37%), whereas DE showed a smaller decrease (−29%), and PL recorded an increase. By contrast, sales were lowest in MT and LU (slightly above 1 tonnes), while in 14 MS, sales represented less than 1% of the EU-27 total.

**Figure 2:**
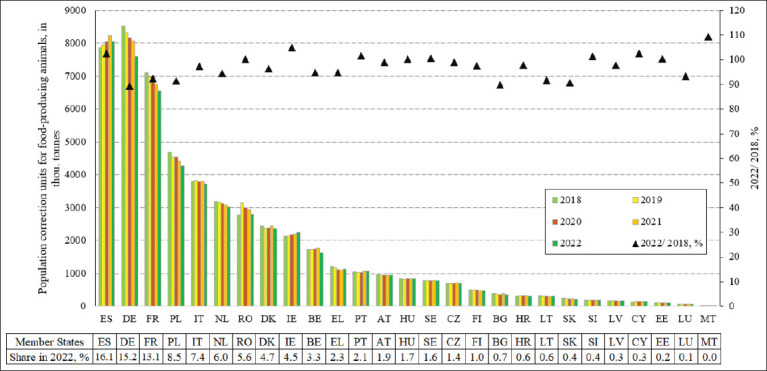
Population correction units for food-producing animals in the European Union Member States in 2018*–*2022, in tonnes, with 2022 share, % and change 2022 is 2018, % [19]. ES = Spain, PL = Poland, IT = Italy, DE = Germany, FR = France, RO = Romania, BE = Belgium, NL = Netherlands, EL = Greece, HU = Hungary, PT = Portugal, DK = Denmark, IE = Ireland, BG = Bulgaria, CY = Cyprus, AT = Austria, CZ = Czech Republic, HR = Croatia, LT = Lithuania, SK = Slovakia, SE = Sweden, FI = Finland, EE = Estonia, SI = Slovenia, LV = Latvia, LU = Luxembourg, MT = Malta.

However, sales data alone do not provide complete insights into progress. Therefore, additional indicators, such as PCUs for food-producing animals, must also be considered to account for the size and composition of the animal population and to compare veterinary AM sales intensity across EU MS. The PCU represents the total biomass of animals potentially treated with AMs (Figure 2) [19].

Although the overall PCU decreased by 4.2% in 2022 compared with 2018 (Figure 2) [19], this reduction was relatively small compared with the sharper decline in sales (Figure 1) [19]. This finding suggests that MS used antibiotics less intensively. The moderate decline in PCU reflected changes in animal population composition, likely due to evolving agricultural structures, management practices, and efforts to mitigate the negative impacts of antibiotic use.

An analysis of total PCUs for the MS with the highest antibiotic sales revealed that they accounted for only 47.2% of the EU-27 total in 2022. ES, PL, and IT showed substantially lower PCU shares compared with their shares of antibiotic sales, indicating intensive antibiotic use. In contrast, DE had a 15.2% share of PCUs but only 12.5% of sales, suggesting less intensive and more efficient usage. Similarly, FR reported 13.1% of PCUs but only 6% of sales, again reflecting effective use.

Importantly, in 10 MS (CY, EE, HU, IE, MT, PT, RO, SI, ES, and SE), PCUs increased while antibiotic sales decreased, highlighting more efficient utilization. At the same time, the decline in PCUs in high-density livestock countries such as DE and FR signaled a positive shift toward less intensive or more efficient production systems.

### Assessment of EU MS performance toward the 2030 F2F target

**Figure 3:**
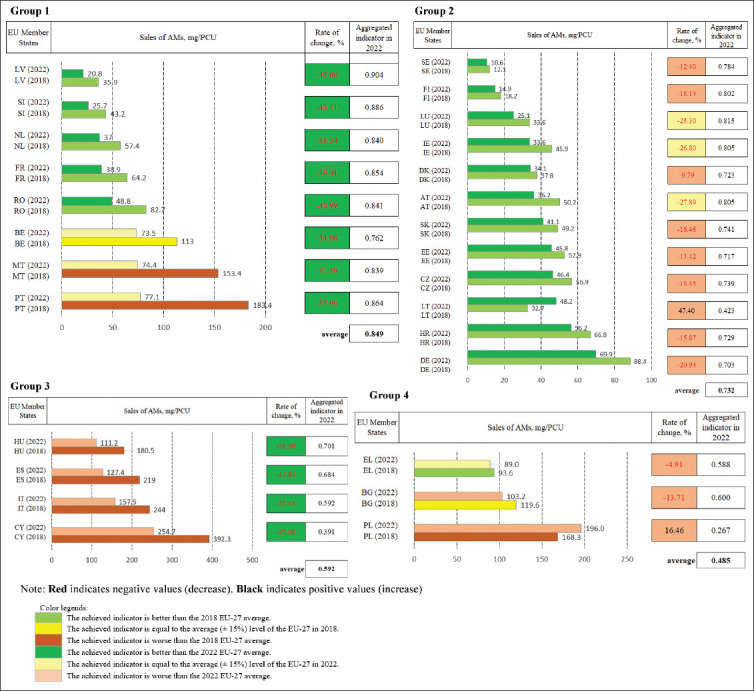
Classification of the European Union Member States by sales of antimicrobials in 2022, mg/population correction unit, and rates of change in 2018–2022, % [19]. ES = Spain, PL = Poland, IT = Italy, DE = Germany, FR = France, RO = Romania, BE = Belgium, NL = Netherlands, EL = Greece, HU = Hungary, PT = Portugal, DK = Denmark, IE = Ireland, BG = Bulgaria, CY = Cyprus, AT = Austria, CZ = Czech Republic, HR = Croatia, LT = Lithuania, SK = Slovakia, SE = Sweden, FI = Finland, EE = Estonia, SI = Slovenia, LV = Latvia, LU = Luxembourg, MT = Malta.

To evaluate current EU-27 progress in achieving the 50% reduction target for AM sales in farmed animals and aquaculture by 2030, MS were grouped into four categories based on indicators and methodology described in the Materials and Methods section (Figure 3) [19].

The data (Figure 3) [19] indicate that veterinary AM sales (mg/PCU) generally improved, with only two MS showing increased sales during the progress period, while all others recorded reductions, albeit at varying rates. Groups 1 and 2 (Figure 3) [19] included MS with sales below the 2022 EU-27 average. In three cases (BE, MT, and PT), sales were close to the EU average (±15%). Rates of change varied between the two groups.

Group 1 (LV, SI, NL, FR, RO, BE, MT, and PT) recorded reductions faster than the EU average, while Group 2 (SE, FI, LU, IE, DK, AT, SK, EE, CZ, LT, HR, and DE) experienced slower-than-average reductions. LT stood out in Group 2, with AM sales increasing by 47.4%. By 2022, MT and PT had already achieved the F2F 50% reduction target, while SI, RO, and LV were approaching this benchmark. The lowest sales volumes were found in SE (10.6 mg/PCU), FI (14.9 mg/PCU), LU (25.1 mg/PCU), LV (20.8 mg/PCU), and SI (25.7 mg/PCU).

Groups 3 and 4 (Figure 3) [19] represented MS with the highest AM sales (mg/PCU). However, Group 3 recorded faster-than-average reductions, while Group 4 decreased sales more slowly than the EU average (-28.3%). In EL, progress was limited (-4.91%), and PL even reported a 16.46% increase. Among these, EL (89 mg/PCU, Group 4) recorded the lowest sales, while CY (254.7 mg/PCU, Group 3) and PL (196 mg/PCU, Group 4) had the highest.

Performance ratings showed Group 1 achieved the strongest contribution (average indicator 0.849), followed by Group 2 (0.732, also strong). Group 3 achieved parity (0.592), while Group 4 showed a limited contribution (0.485). Overall, the EU had achieved more than half of the 2030 reduction target within the first 4 years of the 12 years (2018–2030).

### Performance assessment in the target period (2023–2030)

To assess MS’ future performance, classification into four groups was based on two criteria: (1) the average annual reduction rate required to reach the target and (2) the CAP SP result indicator R.43 (limiting AM use).

- Group 1 (Table 1) [19, 22] included MS that can reduce their average annual reduction rates in 2023–2030 compared with the progress period and have high R.43 values (>50%), indicating ambitious CAP SP commitments. These States have a high probability of achieving the F2F target. MT is notable, having already reached the target during the progress period.
- Group 2 (Table 2) [19, 22] included MS that must maintain or slightly increase reduction rates, with R.43 values at or above the EU average. FI set the highest target (98.1%), though achieving this was considered difficult.
- Group 3 (Table 3) [19, 22] included MS with below-average R.43 values (23.2%) but more modest reduction requirements, making their achievement “probable” or “feasible.” PT, which already achieved the target in the progress period, is notable.
- Group 4 (Table 4) [19, 22] included MS needing to maintain or increase reductions, but with low or average R.43 values, making their achievement uncertain, difficult, or improbable.

Overall, Group 1 was rated as a strong contributor, Groups 2 and 3 as limited, and Group 4 as insufficient.

**Figure 4:**
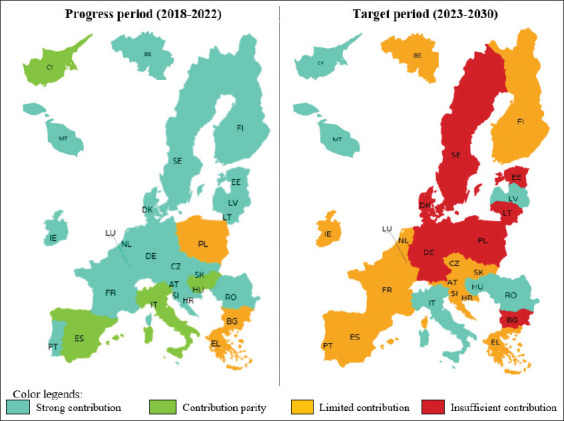
Spatial summary of the European Union Member States’ contributions to achieving the objective of a 50% reduction in the sales of antimicrobials for farmed animals and in aquaculture by 2030 during the progress period and the target period [Source: The map was generated with Piktochart; https://piktochart.com]. ES = Spain, PL = Poland, IT = Italy, DE = Germany, FR = France, RO = Romania, BE = Belgium, NL = Netherlands, EL = Greece, HU = Hungary, PT = Portugal, DK = Denmark, IE = Ireland, BG = Bulgaria, CY = Cyprus, AT = Austria, CZ = Czech Republic, HR = Croatia, LT = Lithuania, SK = Slovakia, SE = Sweden, FI = Finland, EE = Estonia, SI = Slovenia, LV = Latvia, LU = Luxembourg, MT = Malta.

**Figure 5:**
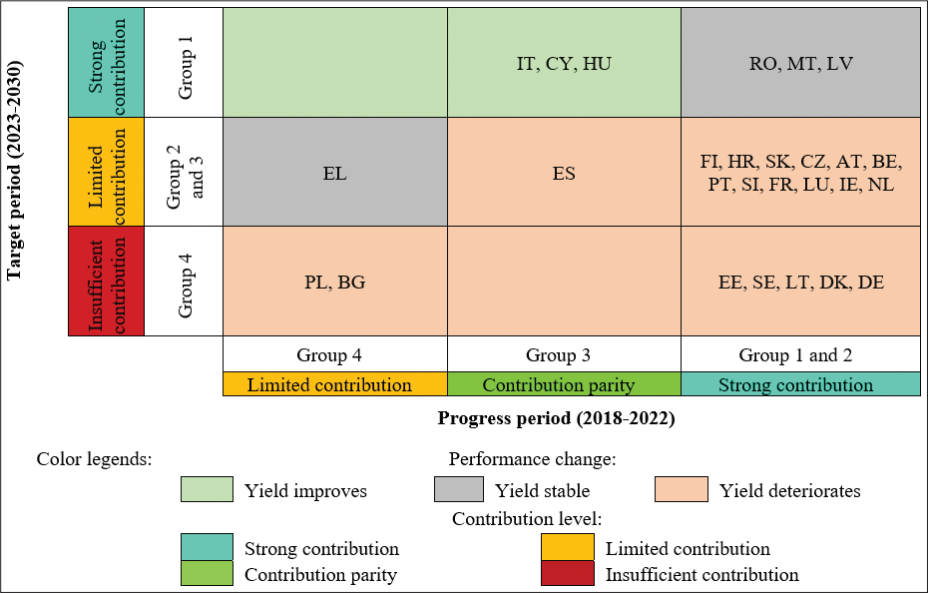
Summary of changes in European Union Member State performance during the progress period and the target period. ES = Spain, PL = Poland, IT = Italy, DE = Germany, FR = France, RO = Romania, BE = Belgium, NL = Netherlands, EL = Greece, HU = Hungary, PT = Portugal, DK = Denmark, IE = Ireland, BG = Bulgaria, CY = Cyprus, AT = Austria, CZ = Czech Republic, HR = Croatia, LT = Lithuania, SK = Slovakia, SE = Sweden, FI = Finland, EE = Estonia, SI = Slovenia, LV = Latvia, LU = Luxembourg, MT = Malta.

Figure 4 provides a spatial summary of MS performance, whereas Figure 5 illustrates changes toward the 50% reduction target by 2030. The summary shows that only three MS (IT, CY, and HU) improved their performance, four (RO, MT, LV, and EL) remained stable, and 20 worsened.

## DISCUSSION

### High-performing EU MS with full or near-target achievement

This research identified two MS, MT and PT, those have already achieved the F2F strategy’s goal of reducing AM sales in farming by 50% by 2030 compared with 2018 levels. Several others, including SI, RO, and LV, are close to reaching this target. Although these countries contribute relatively little to overall EU sales because of their smaller agricultural land areas and herd sizes, they provide valuable models for successful AM reduction strategies. For this reason, the experience of PT and MT should be analyzed in greater depth, as both demonstrate effective policies that can serve as precedents for other MS in curbing AMR and promoting sustainable livestock practices.

PT has implemented stringent controls and surveillance mechanisms for veterinary AM use, supported by a One Health policy framework. Prescribing behaviors and AM practices of veterinarians are closely monitored [32]. However, PT could further strengthen its approach by incorporating additional best practices, such as Dutch-style farm-level decision-making tools [33]. MT has also achieved the reduction target, albeit with low absolute quantities. Looking ahead, RO, LV, and SI appear to be the “future leaders.” RO reported 136 tonnes of AMs marketed in 2022 and is on track toward the target, while LV and SI have made consistent progress through heightened awareness, targeted veterinary controls, and integrated animal health policies [34]. In all these countries, strategic veterinary oversight combined with policy incentives has been instrumental [35].

### Rapid reductions and strategic initiatives by EU MS

In 2022, the NL, FR, and BE recorded faster-than-average reductions in AM sales between 2018 and 2022 (Figure 1) [19]. The NL and FR were pioneers in implementing farm-level monitoring [33], benchmarking systems, and bans on critically important antibiotics [36]. FR further integrated AMs prescription control into national animal health law as early as 2015 [37]. Strong public pressure from civil society and consumer organizations reinforced these measures [38]. In both BE and the NL, the establishment of national targets spurred the development of comprehensive national strategies to reduce AMs use in animals [10].

DK and SE also imposed restrictions early on and have since built on robust national stewardship programs [10]. SE, in particular, has historically recorded among the lowest veterinary AMs use in the EU, a result of strict prescription rules and close cooperation between veterinarians and farmers [38].

### EU MS as strong contributors without explicit national targets

DE and IE provide key examples of countries that have achieved satisfactory reductions in AM sales without adopting explicit national targets [10]. In IE, emphasis is placed on communicating best practices to farmers to enhance animal health and thereby reduce the need for antibiotics [10]. Both DE and IE can be categorized as strong contributors to the F2F AMs reduction goal, a group that also includes the NL, BE, FR, PT, MT, LV, RO, DK, and SE.

### EU MS with rising AM sales and required policy shifts

By contrast, PL and LT recorded increases in AM sales (mg/PCU) during the period of 2018–2022. These countries are under immediate pressure to adopt proven best practices from their peers. In PL, the endemic use of AMs in cattle herds has highlighted the urgent need for stronger policy and educational interventions aimed at more effective disease control and herd management [39]. In LT, the EMA (2023) noted that although a national system for collecting AMs use data exists, it is not yet fully functional to ensure comprehensive monitoring and reporting [40].

### Scientific and policy recommendations

Future studies should conduct in-depth investigations into AMR and strategies for reducing antibiotic use, particularly those with practical applications in cattle production [41]. Research and development must also be directed toward discovering alternative antibiotics [42], alongside the introduction of new diagnostic technologies to enable rapid and accurate infection detection and thus minimize unnecessary antibiotic use [43]. Regular reviews of the evidence base on AM treatments are necessary, as well as expanded research to evaluate treatment practices under diverse farm-level conditions [44].

Given that this review relies on secondary evidence (EMA data), limitations include a lack of regional or farm-specific granularity and assumptions embedded in prediction models, which reduce the precision of performance groupings. Future analyses should incorporate farm-level data, assess AMR trends in detail, and evaluate the real-world effectiveness of implemented policies.

MS must align national policies with EU-wide targets through measures such as benchmarking, prescription control, and AMS interventions. Cross-country cooperation is also vital to facilitate the exchange of best practices and effective strategies for reducing AMs use [45] while safeguarding animal health and welfare [46]. In some MS, it may be necessary to design comprehensive national AM policies with appropriate technical, legal, economic, and policy resources [47]. Stronger management of AMs will also contribute to improved biosecurity and production outcomes [48].

Veterinarians must continue to receive education on all aspects of AMR and AMs treatment to ensure rational use of antibiotics and to minimize AMR risks for both animals and humans [49]. Finally, advancing cross-sectoral collaboration between veterinary, human health, and environmental domains remains essential for addressing AMR holistically.

The authors emphasize the need for continued, regular assessment of EU-27 performance in meeting the F2F target of reducing AM sales in agriculture by 50% by 2030 compared with 2018 levels. Monitoring of AM sales and resistance trends at the EU scale should remain in place, with harmonized indicators extended even beyond 2030.

## CONCLUSION

This study provides a comprehensive review of the performance of EU MS against the European Green Deal’s F2F strategy target to reduce AM sales in agriculture by 50% by 2030. Findings reveal varied performance among the EU-27 countries, with some reporting substantial improvement, while others are at risk of failing. Overall, the trend shows a decline in AM sales from 2018 to 2022, but with uneven rates of reduction, differing policy commitments, and disparate CAP SP targets, suggesting the need for more specific and concerted action. The most recent decreases were reported in PT, MT, FR, LV, RO, and ES, while LT and PL recorded increases. Notably, the four largest markets–ES, PL, IT, and DE–account for 70% of total sales, thereby playing a pivotal role in meeting the EU-wide target.

The analysis of PCUs revealed a slight overall decrease in the EU from 2018 to 2022, lagging behind the sharper drop in antibiotic sales. This indicates a trend toward more efficient AMs use. For the four highest-sales MS (ES, PL, IT, and DE), total PCUs represented 47.2% of the EU-27 total, highlighting relatively intensive antibiotic use in ES and PL. In contrast, DE and FR displayed less intensive and more effective AM practices. In 10 EU MS, total PCUs increased while AM sales declined, suggesting improved efficiency, whereas declines in both PCUs and sales in high-animal-density states (e.g., DE and FR) may indicate shifts toward more sustainable production.

Between 2018 and 2022, AM sales (mg/PCU) declined in almost all EU MS, except PL and LT, which saw increases. Some countries, including MT and PT, have already surpassed the 50% reduction target, with LV also close. SE, FI, LU, and LV reported the lowest sales levels. Performance assessments during the progress period (2018–2022) rated 20 MS as “strong contributors,” four (HU, ES, IT, and CY) as “contribution parity,” and three (EL, BG, and PL) as “limited contributors.”

Looking ahead to the target period (2023–2030), several MS may experience slower reductions in AM sales. While MT and FI have set ambitious goals, other MS must intensify efforts. Six MS (RO, IT, MT, LV, CY, and HU) are projected to remain “strong contributors,” five (FI, EL, HR, SK, and CZ) will remain “limited,” and nine (AT, BE, PT, SI, FR, LU, ES, IE, and NL) are rated below average. In contrast, seven MS (EE, PL, BG, SE, LT, DE, and DK) are expected to underperform, being rated “insufficient.”

A comparative assessment of the two periods showed that only three MS (IT, CY, and HU) improved their performance, four (RO, MT, LV, and EL) remained stable, while 20 deteriorated. These findings highlight the urgent need for MS with lagging progress to revise CAP SP ambitions, strengthen veterinary and farming incentives to reduce AMs use, and improve monitoring mechanisms. EU-wide progress can be accelerated through greater exchange of best practices from high-performing MS.

This study is not without limitations. Reliance on secondary EMA data masks regional and farm-level variations, while projections for target periods rest on assumptions about reduction rates and policy effectiveness that may not align with on-the-ground realities.

Future research should incorporate farm-level disaggregated data to validate the effectiveness of AM reduction policies, examine the relationship between reduced AMs use, animal health outcomes, and AMR dynamics, and explore long-term consequences of AMs reduction, such as shifts in disease incidence or implications for animal welfare.

## AUTHORS’ CONTRIBUTIONS

AM: Conceptualization and validation, methodology, investigation, visualization, data analysis, formal analysis, and drafted the manuscript. IP: Conceptualization and validation, methodology, investigation, data analysis, and drafted the manuscript. IU: Investigation, data analysis, and edited the manuscript. KM: Visualization and software. AN: Project administration and supervision. All authors have read and agreed to the published version of the manuscript.
